# Identification of Transcriptional Pattern Related to Immune Cell Infiltration With Gene Co-Expression Network in Papillary Thyroid Cancer

**DOI:** 10.3389/fendo.2022.721569

**Published:** 2022-02-04

**Authors:** Meiye Li, Jimei Zhang, Zongjing Zhang, Ying Qian, Wei Qu, Zhaoshun Jiang, Baochang Zhao

**Affiliations:** ^1^ Department of Endocrinology, No. 960 Hospital of PLA Joint Logistics Support Force, Jinan, China; ^2^ School of Pharmacy, Shandong First Medical University & Shandong Academy of Medical Sciences, Taian, China; ^3^ School of Life Sciences, Shandong First Medical University & Shandong Academy of Medical Sciences, Taian, China

**Keywords:** biomarkers, immune cell infiltration, weighted gene co-expression network, papillary thyroid cancer, The Cancer Genome Atlas database

## Abstract

**Background:**

A growing body of evidence suggests that immune cell infiltration in cancer is closely related to clinical outcomes. However, there is still a lack of research on papillary thyroid cancer (PTC).

**Methods:**

Based on single-sample gene set enrichment analysis (SSGSEA) algorithm and weighted gene co-expression network analysis (WGCNA) tool, the infiltration level of immune cell and key modules and genes associated with the level of immune cell infiltration were identified using PTC gene expression data from The Cancer Genome Atlas (TCGA) database. In addition, the co-expression network and protein-protein interactions network analysis were used to identify the hub genes. Moreover, the immunological and clinical characteristics of these hub genes were verified in TCGA and GSE35570 datasets and quantitative real-time polymerase chain reaction (PCR). Finally, receiver operating characteristic (ROC) curve analysis was used to evaluate the diagnostic value of hub genes.

**Results:**

Activated B cell, activated dendritic cell, CD56bright natural killer cell, CD56dim natural killer cell, Eosinophil, Gamma delta T cell, Immature dendritic cell, Macrophage, Mast cell, Monocyte, Natural killer cell, Neutrophil and Type 17 T helper cell were significantly changed between PTC and adjacent normal groups. WGCNA results showed that the black model had the highest correlation with the infiltration level of activated dendritic cells. We found 14 hub genes whose expression correlated to the infiltration level of activated dendritic cells in both TCGA and GSE35570 datasets. After validation in the TCGA dataset, the expression level of only 5 genes (C1QA, HCK, HLA-DRA, ITGB2 and TYROBP) in 14 hub genes were differentially expressed between PTC and adjacent normal groups. Meanwhile, the expression levels of these 5 hub genes were successfully validated in GSE35570 dataset. Quantitative real-time PCR results showed the expression of these 4 hub genes (except C1QA) was consistent with the results in TCGA and GSE35570 dataset. Finally, these 4 hub genes had diagnostic value to distinguish PTC and adjacent normal controls.

**Conclusions:**

HCK, HLA-DRA, ITGB2 and TYROBP may be key diagnostic biomarkers and immunotherapy targets in PTC.

## Introduction

Thyroid cancer is the most common endocrine malignancy, accounting for about 3% of the total incidence of all malignancies, and its incidence is growing rapidly worldwide (1). Based on the pathological classification, thyroid cancer can be divided into papillary thyroid, follicular thyroid, medullary thyroid and undifferentiated thyroid cancer types (2). About 83% of patients with thyroid cancer belong to papillary thyroid carcinoma (PTC). Although PTC has a low mortality rate, it has a high rate of recurrence or progression, which places a heavy financial and emotional burden for patients with PTC (3). Currently, the treatment strategies of PTC have made great progress, but they still can’t fully ameliorate the survival with locally advanced or distant metastatic PTC. Thence, it is necessary to further understand the pathogenesis of PTC and discover novel therapeutic targets.

Tumor-infiltrating immune cells have been reported to help the host resist cancer cells proliferation and invasion, and accelerate the solid tumors initiation and development. The thyroid, the largest endocrine organ, is a common target of autoimmune diseases. It has been reported that the autoimmune disease Hashimoto’s disease may drive and promote the PTC progression (4, 5). Furthermore, immune cells have been reported to be generally found in the thyroid tumor microenvironment and to form different tumor microenvironments at different stages of tumor progression (6). It is reported that the changes of immune cell infiltration components have become an important predictor of the survival of various solid tumors (7). The infiltration levels of immune cells in PTC microenvironment are significantly abnormal (8, 9). High expression of claudin 10 (CLDN10) may improve the outcome of patients with PTC by increasing immune cell infiltration (including CD8 T cells and macrophages) (10). However, the specific mechanism of PTC immune cell infiltration is unclear. Therefore, a systematic understanding of the correlation between PTC gene and immune cell infiltration is of great significance for subsequent research and treatment.

As a bioinformatics new approach, Single-sample Gene Set Enrichment Analysis (SSGSEA) has been used to uncover the level of immune cell infiltration in a variety of cancer cells (11). SSGSEA defined the immune cell-related gene set, and characterized density of tumor infiltrated immune cells by scoring. Researchers can calculate the characteristics of tumor immune cell infiltration in tumor microenvironment by SSGSEA algorithm, rather than by immunohistochemistry and flow cytometry (12). Weighted gene co-expression network analysis (WGCNA) is a commonly used bioinformatics tool for effectively studying the correlation between genes and phenotypes (13). Based on the weighted co-expression, the correlation between gene expression in different samples can be described, and the candidate target genes can be finally screened, suggesting that WGCNA provides a new way for us to study disease characteristics and identify phenotypic related genes (14, 15).

To reveal the role of immune cells infiltration level in PTC, levels of the immune cell infiltration in PTC and normal tissue samples were assessed by SSGSEA algorithm. Then, the WGCNA tool was used to identify key modules and genes associated with the level of immune cell infiltration. Ultimately, the immunological and clinical characteristics of these genes were demonstrated using database analysis and quantitative real-time polymerase chain reaction (PCR). Our work will contribute to the understanding of the molecular mechanisms of immune cell infiltration in PTC.

## Materials and Methods

### Data Preprocessing

Case information of PTC, concerning the gene expression transcripts per million (TPM) values and clinical features, were obtained from The Cancer Genome Atlas (TCGA) database, which was obtained from the University of California Santa Cruz Xena (UCSC Xena; https://xena.ucsc.edu/) database. Data of 501 PTC and 58 adjacent normal samples were used for training dataset. The expression matrix of GSE35570 dataset containing 65 PTC patients and 51 adjacent normal samples was downloaded from Gene Expression Omnibus (GEO) database (http://www.ncbi.nlm.nih.gov/geo/) (16), and extracted for validation dataset.

### Evaluation of Immune Cell Infiltration

Firstly, from Charoentong’s research, a set of genes that mark each infiltrating immune cell type was obtained (17, 18). Then, SSGSEA algorithm was used to obtain the differences of immune cell infiltration between PTC and adjacent normal groups (19). The Wilcoxon test was used to calculate the significance of differences in immune cell infiltration. Enrichment scores calculated by SSGSEA analysis were used to represent the relative abundance of immune infiltrated cells in each tissue sample. The box plots compare the differences in the level of immune cells infiltration in different sample tissues. In addition, the correlation between immune cells was calculated using Pearson correlation analysis.

### Weighted Gene Co-Expression Network Construction and Identification of Hub Modules

The “WGCNA” package in R (http://www.r-project.org/) was used for WGCNA (20). To uncover the relationship between functional modules and immune cell infiltration in PTC patients, we selected the top 25% variance genes to perform the WGCNA. Firstly, based on the Pearson correlation between paired genes, the expression level of a single transcript was converted into a similarity matrix and converted to the adjacency matrix (13). When soft-thresholding power was 6, the adjacency matrix was then converted to a topological overlap matrix. In order to classify genes with similar expression patterns into different modules, the dynamic hybrid cutting method was adopted, and the minimum module size cut-off value was 30 (21, 22).

### Identification of Clinical Significant Modules and Enrichment Analysis

Component analysis of each module was carried out by module characteristic genes. We calculated the correlation between the characteristic genes of modules and immune cell infiltration cells by Pearson test to determine the significance of modules. The cut-off of the co-expression module was set to P < 0.05. Then, we further calculated and visualized the differences of module characteristic genes, selected a cutting line for the module tree and merged some modules. We selected interested immune cells and the modules with the highest correlation, and defined them as hub modules. To further study the biological function of genes in the hub module, the David6.8 (https://david.ncifcrf.gov/home.jsp) was used for gene ontology (GO) analysis and the kyoto encyclopedia of genes and genomics (KEGG) pathway enrichment analysis.

### Identification and Verification of Hub Genes

We selected candidate hub genes according to the module connectivity and clinical traits of each gene in hub module. Module connectivity is defined as the absolute value of Pearson correlation between genes (Module Membership; MM). The relationship between clinical traits is defined as the absolute value of Pearson correlation between genes and traits (Gene Significance; GS). In hub module, we selected the genes with MM > 0.8 and GS > 0.8 as candidate hub genes. At the same time, the candidate hub genes were used to construct protein-protein interaction (PPI) network by string database (https://string-db.org/). Cytoscape software (http://www.cytoscape.org) was used to visualize the network. The genes with Degree > 15 was considered as hub genes. The correlation between hub gene and Activated dendritic cell was proved by SSGSEA algorithm in GSE35570 dataset. In addition, the correlation between hub gene and Activated dendritic cell was also calculated in the TISIDB database (http://cis.hku.hk/TISIDB/index.php).

### Identification of Immune and Clinical Features

Spearman correlation between different immune factors and hub gene was obtained from TISIDB database, which includes immunosuppressive factors and immune stimulating factors, chemokines and receptors (23). Then, we used R package heatmap to construct the heat map. Cytoscape was used to select hub genes with average correlation coefficients greater than 0.5 and related immune factors to construct the network. To explore the clinical features of these hub genes, the R package limma was used to analyze the differences in all genes, and the R package ggplot2 was used to map volcano. The statistical significance between normal samples and tumor samples was analyzed using wilcoxon rank sum test. The GSE35570 dataset was also used to verify the expression of hub genes.

### *In Vitro* Validation

Six tumor and matched adjacent normal tissues were obtained from PTC patients with written consent, which was approval by the ethics institute of our hospital. The tissues were flash frozen for later testing. All the patients were aged between 32 to 51 years old. Detailed inclusion criteria for patients with PTC were as follows: (1) patients were initially diagnosed with PTC by histopathological or cytological examination; (2) patients had no history of other malignant tumors; (3) patients had not been treated with radiation or chemotherapy before diagnosis; (4) patients had no other autoimmune diseases. Total RNA was isolated using a TRIzol reagent (Life Technologies, Carlsbad, CA). Complementary DNA reverse transcription, and quantitative real-time PCR were performed by ReverTra Ace qPCR RT Kit (Toyobo, Osaka, Japan) and Super Real PreMix Plus SYBR (Toyobo, Osaka, Japan), respectively. The human GAPDH and actin were used as endogenous controls for gene expression in analysis. Fold change was calculated with the 2^−ΔΔCt^ formula normalized to five genes (24). According to log 2 fold change > 0 or log 2 fold change < 0, it can be clearly seen whether the gene was up-regulated or down-regulated. In addition, tumor and adjacent normal tissue samples were obtained for immunohistochemical analysis. We randomly selected 2 genes from hub genes for verification. Brown-yellow coloring was defined as positive expression.

For the results from the quantitative real-time PCR on hub genes expression, data was presented as mean ± SD. Means between two groups were compared using the Test. P < 0.05 was considered to be statistically significant.

### Receiver Operating Characteristic (ROC) Curve Analyses

To evaluate the diagnostic value of hub genes, the “pROC” package was performed to generate ROC, and the area under the ROC curve (AUC) represented the diagnostic value. The sensitivity and specificity at the cut-offs were determined according to a previous study (25). When AUC value was greater than 0.7, the hub gene was considered to be able to distinguish between PTC and adjacent normal with good specificity and sensitivity (26–28).

### Statistical Analysis

R software was used for all statistics (version 4.0.0; https://www.R-project.org). The Wilcoxon test was used to calculate the significance of differences in immune cell infiltration and gene expression. The correlation between immune cells was calculated by Pearson correlation analysis. Relative gene expression was analyzed using the 2^−ΔΔCt^ method. All experiments were independently repeated at least three times. One-way analysis of variance method was used for quantitative real-time PCR validation experiment. Mean ± SD was used to show the results. P < 0.05 was considered statistically significant.

## Results

### Evaluation of Immune Cell Infiltration

**Figure 1:**
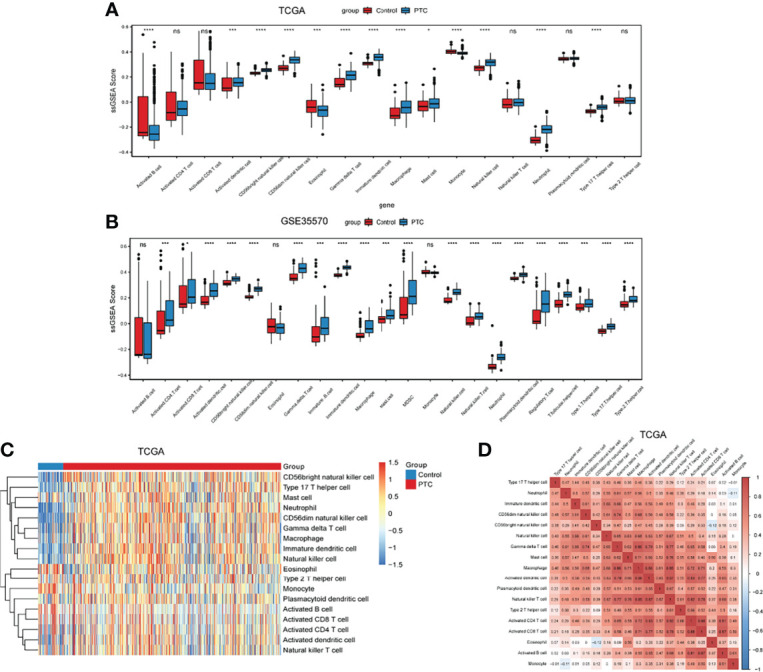
The level of immune infiltration cells between PTC and adjacent normal controls. **(A)** The distribution of immune infiltration cells between PTC and adjacent normal controls in TCGA. The x-axis shows the different immune cells, and the y-axis shows the algorithm score of SSGSEA. *P < 0.05; ***P < 0.001; ****P < 0.0001. P < 0.05 is considered statistically significant. ns represent no statistical significance. **(B)** The distribution of immune infiltration cells between PTC and adjacent normal controls in GSE35570 dataset. The x-axis shows the different immune cells, and the y-axis shows the algorithm score of SSGSEA. ***P < 0.001; ****P < 0.0001. P < 0.05 was considered statistically significant. ns represent no statistical significance. **(C)** Heat maps of immune cells between PTC and adjacent normal. **(D)** Correlation analysis of immune cells between PTC and adjacent normal. X-axis and Y-axis represents the above immune cells. Number represents the correlation. A larger number implies a higher correlation.

The differential of immune cell infiltration between PTC and adjacent normal tissues were evaluated by SSGSEA algorithm. The distribution of immune cell infiltration cells between PTC and adjacent normal tissues is exhibited in **Figure 1A**. We found that Activated B cell, Activated dendritic cell, CD56bright natural killer cell, CD56dim natural killer cell, Eosinophil, Gamma delta T cell, Immature dendritic cell, Macrophage, Mast cell, Monocyte, Natural killer cell, Neutrophil and Type 17 T helper cell were significantly changed between PTC and adjacent normal groups, while Activated CD4 T cell, Activated CD8 T cell, Natural killer T cell, Plasmacytoid dendritic cell and Type 2 T helper cell were not significantly altered between PTC and adjacent normal groups. Among which, Activated dendritic cell, CD56bright natural killer cell, Gamma delta T cell, Immature dendritic cell, Macrophage, Mast cell, Natural killer cell, Neutrophil and Type 17 T helper cell were obviously increased and Activated B cell, CD56dim natural killer cell, Eosinophil and Monocyte were markedly decreased in PTC groups compared with adjacent normal groups. The gene lists in immune cells obtained from Charoentong’s research (29) are listed in **Supplementary Table S1**. In addition, we verified the distribution of immune cell infiltration cells between PTC and adjacent normal tissues in the GSE35570 validation dataset. The result in the validation dataset was highly consistent with the result in the training dataset (**Figure 1B**). Heat maps of immune cells between PTC and adjacent normal is presented in the **Figure 1C**. The correlation analysis of immune cells is shown in **Figure 1D** and **Supplementary Table S2**. These results suggested that above immune cells may be involved in the progression of PTC.

### Weighted Gene Co-Expression Network Construction and Identification of Hub Modules

**Figure 2:**
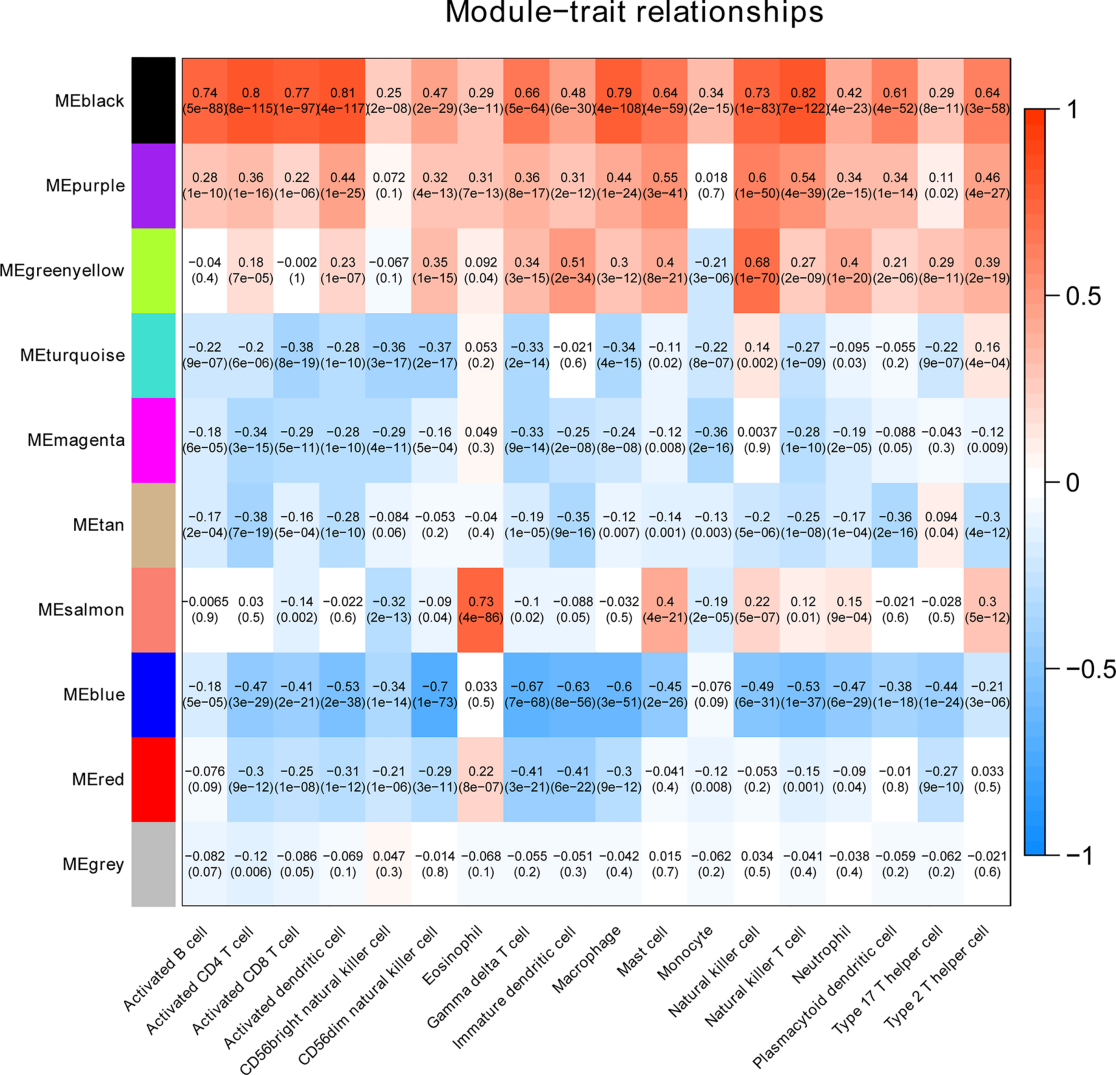
The heatmap shows the correlation between module characteristic genes and the immune cell infiltration of PTC.

**Figure 3:**
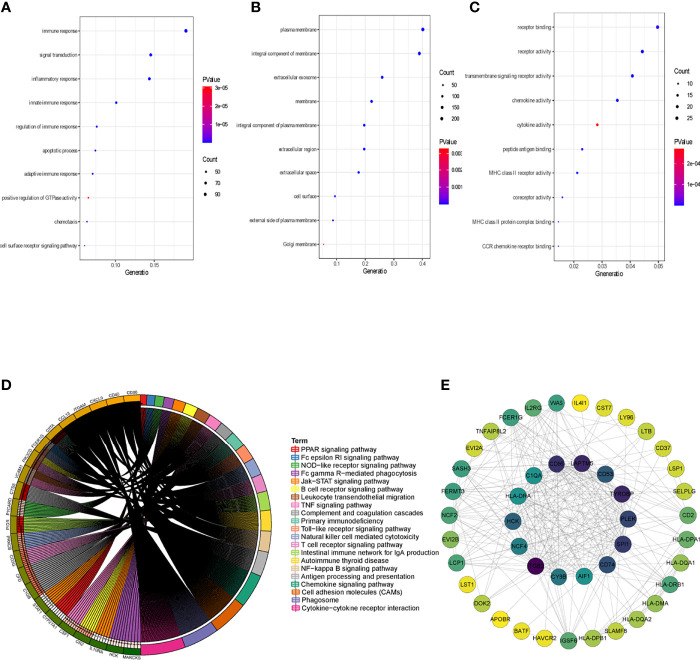
Identification of hub genes. **(A)** Top 10 significantly enriched GO terms: Biological process (BP) enrichment of the black module genes. **(B)** Top 10 significantly enriched GO terms: cellular component (CC) enrichment of the black module genes. **(C)** Top 10 significantly enriched GO terms: molecular function (MF) enrichment of the black module genes. **(D)** KEGG pathway enrichment in black module genes. **(E)** PPI network of genes from the black module. The higher number of connected nodes represents the larger size of the node.

To clarify the association between functional modules and immune cell infiltration in patients with PTC, we selected the 25% of genes with the greatest difference, for WGCNA. The sample dendrogram and trait heatmap are displayed in **Supplementary Figure 1A**. In order to build scale-free network, the soft threshold cut-off standard power with power β = 6 was selected (**Supplementary Figure 1B**). Dynamic tree cutting method was applied to merge modules with dissimilarity less than 30%, and 10 modules were finally obtained (**Supplementary Figures 2A, B**). Correlation analysis between the eigengenes of each module and immune cell is presented in **Figure 2**. Compared with other modules, black module was most associated with the level of infiltration of immune cells. All genes in the black module are shown in **Supplementary Table S3**. In addition, compared with other immune cells, the relationship between Activated dendritic cells and PTC is less studied. In this study, the black module was highly related to Activated dendritic cells. Thence, we selected black module and Activated dendritic cells for further analysis. Intra module analysis was carried out on the black module. There was a very significant correlation between module membership and gene significance (cor =0.88, p =1.3E-180), suggesting that 554 genes in the black module tended to be significantly correlated with infiltration level of Activated dendritic cells (**Supplementary Figure 2C**). For this reason, the black module was considered to be PTC-related hub module. To illustrate the affected functions of the genes clustered in the black module, the GO and KEGG analysis was further performed using the David6.8. Based on the GO enrichment analysis, immune response, signal transduction, plasma membrane, integral component of membrane, receptor binding and receptor activity were significantly enriched GO terms (**Figures 3A**–**C**). The KEGG pathway enrichment analysis revealed that most genes were mainly enriched in pathways including cytokine-cytokine receptor interaction, cell adhesion molecules (CAMs), phagosome, chemokine signaling pathway, antigen processing and presentation, autoimmune thyroid disease (a common autoimmune disease in clinical, and the diseased organ is the thyroid gland), etc. (**Figure 3D**).

### Identification and Validation of Hub Genes

**Figure 4:**
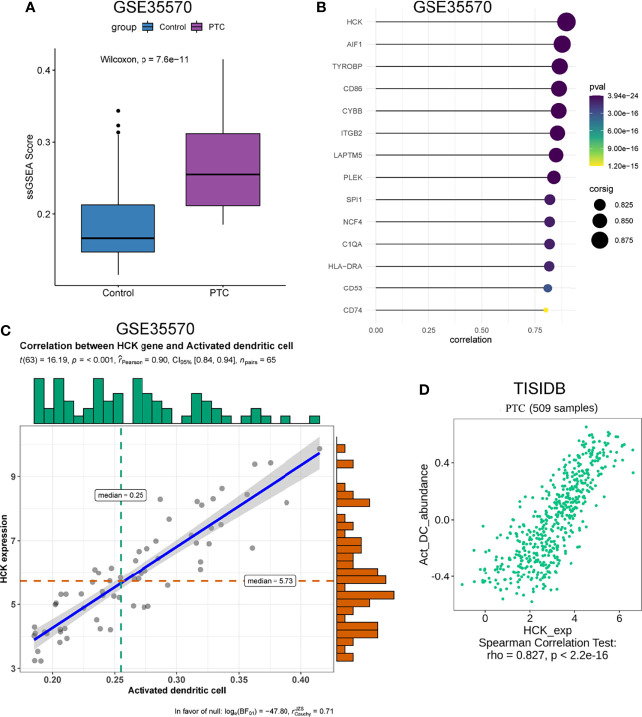
Validation of 14 hub genes. **(A)** The infiltration level of activated dendritic cells level between PTC and adjacent normal groups. **(B)** Relationship between 14 hub genes expression and activated dendritic cells infiltration level. **(C)** Correlation analysis of HCK expression and the infiltration level of activated dendritic cells based on GSE35570 dataset. n represents the number of PTC samples in the dataset. **(D)** Correlation analysis of HCK expression and the infiltration level of Activated dendritic cells in TISIDB database.

The genes that were highly linked to the black module and associated with Activated dendritic cells infiltration level were studied. Based on the cut-off threshold (MM > 0.8 and GS > 0.8), a total of 45 genes were identified as candidate hub genes (**Supplementary Figure 2C**). We then performed the PPI network of genes in black module, genes with Degree>15 were identified to be the central nodes. We obtained 14 central nodes and visualized these results using Cytoscape (**Figure 3E**). To verify the relationship between these 14 hub genes and Activated dendritic cells, we used GSE35570 dataset to uncover the Activated dendritic cells infiltration level, and found that the Activated dendritic cells level was significantly increased between PTC and adjacent normal groups, which is consistent with our analysis in the TCGA dataset (**Figure 4A**). Spearman correlation analysis results in GSE35570 dataset showed positive correlation of the expression values of the 14 genes with the Activated dendritic cells infiltration level (correlation coefficient of > 0.75; **Figure 4B**). For instance, in **Figures 4C, D**, we showed a scatter plot of **HCK** expression and Activated dendritic cells infiltration level in GSE35570 dataset and TISIDB database. In addition, we further verify the correlation between 14 genes and Activated dendritic cells infiltration level in GSE33630. The result again proved that 14 genes were significantly correlated with Activated dendritic cells infiltration level (**Supplementary Figure 2D**). C1QA is listed as a key gene, but this is more often associated with macrophages; NCF4 is a part of the oxidative burst genes in phagocytes. CD53 and HCK are broadly expressed across myeloid populations. AIF1 is associated with macrophage activation, and some lymphocyte migration (including T cells). In comparison CD86, CD74, and HLA-DRA are more specific to antigen presenting cells. Although these hub genes were not the specific genes most associated with activated dendritic cells, they were significantly associated with activated dendritic cells in PTC by correlation analysis. In summary, the identified central genes may be highly correlated with the level of Activated dendritic cells infiltration, and these genes may play a key role in the development of PTC. In addition, these results provide potential research directions for further study of the pathological mechanism of PTC.

### Identification of Immune and Clinical Features

**Figure 5:**
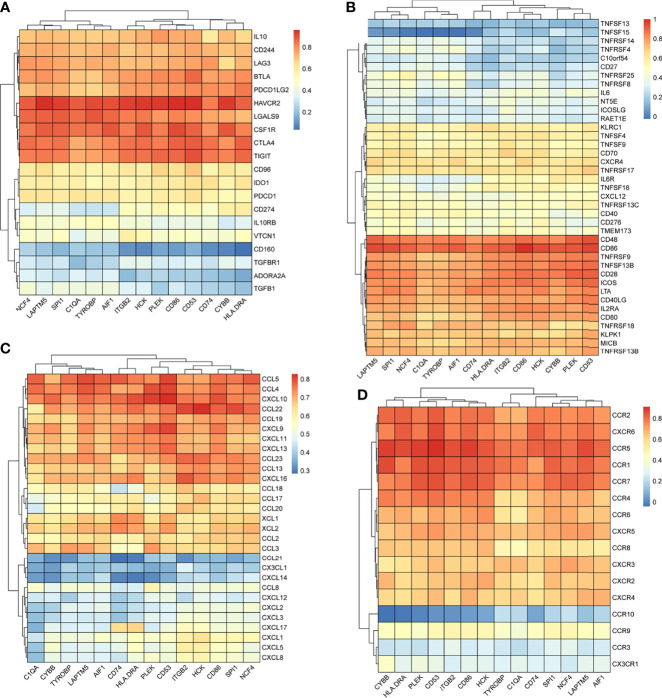
The spearman correlation between the expression of 14 hub genes and the expression of immune factors in the TISIDB database. **(A)** Immunosuppressor factors. **(B)** Immunostimulator factors. **(C)** Chemokines. **(D)** Receptors.

**Figure 6:**
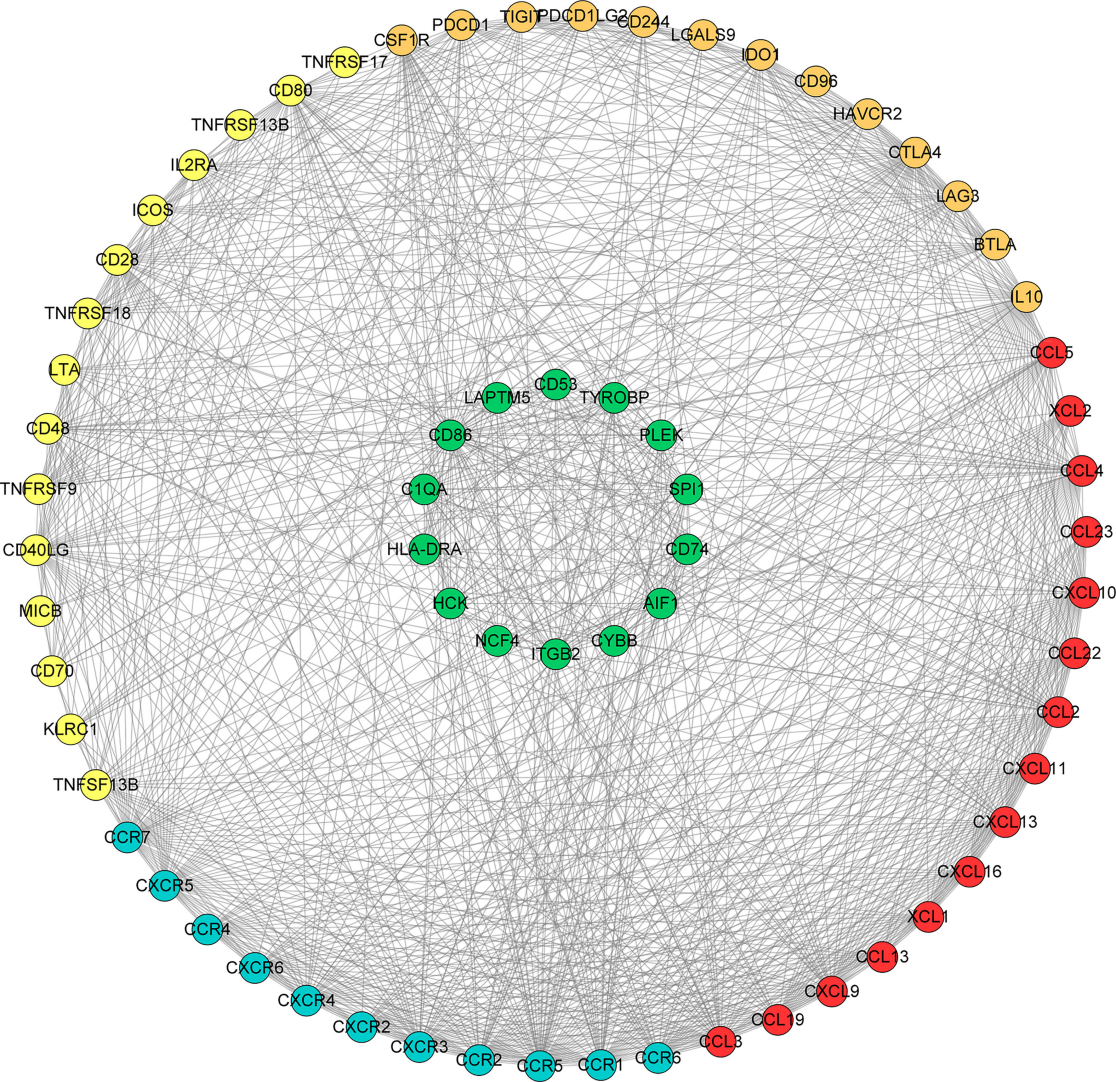
Interaction network between hub genes and immune factors based on 14 hub genes and 54 immune-related factors. Orange, yellow, red and blue color represents immunosuppressive factor, immune stimulating factor, chemokine and receptor, respectively. The green colors indicate 14 hub genes.

**Figure 7:**
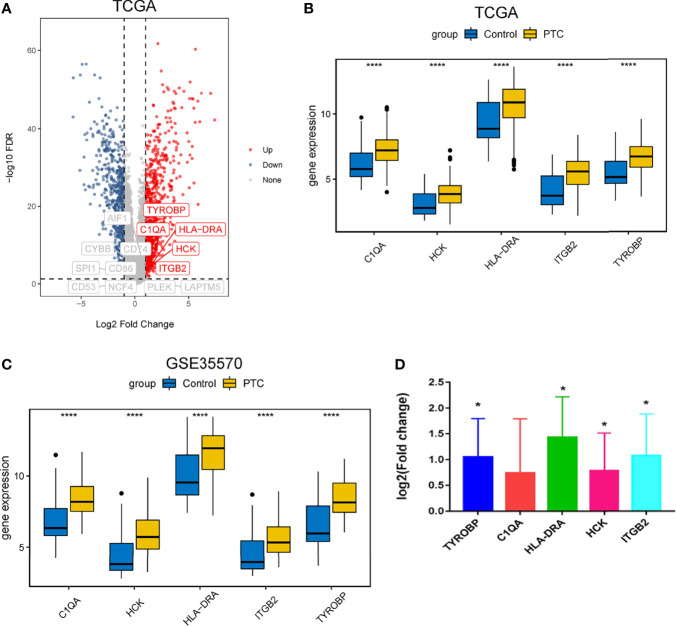
Expression validation of hub genes. **(A)** Volcanic maps of hub genes in TCGA dataset. **(B)** Expression level of hub genes was analyzed based on TCGA dataset. ****P < 0.0001. **(C)** Expression level of hub genes was analyzed based on GSE35570 dataset. ****P < 0.0001. **(D)** Expression level of hub genes was measured by quantitative real-time PCR. Log 2 fold change > 0, up-regulation; log 2 fold change < 0, down-regulation; *P < 0.05. P < 0.05 was considered statistically significant.

We searched the spearman correlation between the expression of these 14 hub genes and the expression of immune factors in the TISIDB database, including immunosuppressor factors, immunostimulator factors, chemokines and receptors. Heat map of correlation is presented in **Figure 5**. The results showed that PTC hub genes were significantly correlated with Th1 cell suppressor factor (HAVCR2), T cell activator factor (CD86), T cell chemokine (CCL5) and macrophage virus receptor (CCR5). In addition, the genes (CD80, CCR7) expressed on Activated dendritic cells were also related to the hub genes. This implies that immune mediation plays an important role in the progression of PTC. We identified 54 immune-related factors with an average correlation of greater than 0.5 for all 14 hub genes. Interaction network between hub genes and immune factors was constructed based on 14 hub genes and 54 immune-related factors (**Figure 6**). Expression validation of hub genes in TCGA was performed, and results suggested that only 5 genes (C1QA, HCK, HLA-DRA, ITGB2 and TYROBP) were differentially expressed between PTC and adjacent normal groups (**Figure 7A**). The boxplots of five genes expression is presented in the **Figure 7B**. Similarly, we also validated expression level of 5 genes in GSE35570 dataset (**Figure 7C**). In addition, we analyzed the correlation between these 5 genes and Activated dendritic cells pre- and post-Chernobyl in the GSE35570 dataset. The results showed the same correlation between Activated dendritic cells and genes pre- and post-Chernobyl (**Supplementary Figure 3**).

### *In Vitro* Validation and ROC Curve Analyses

**Figure 8:**
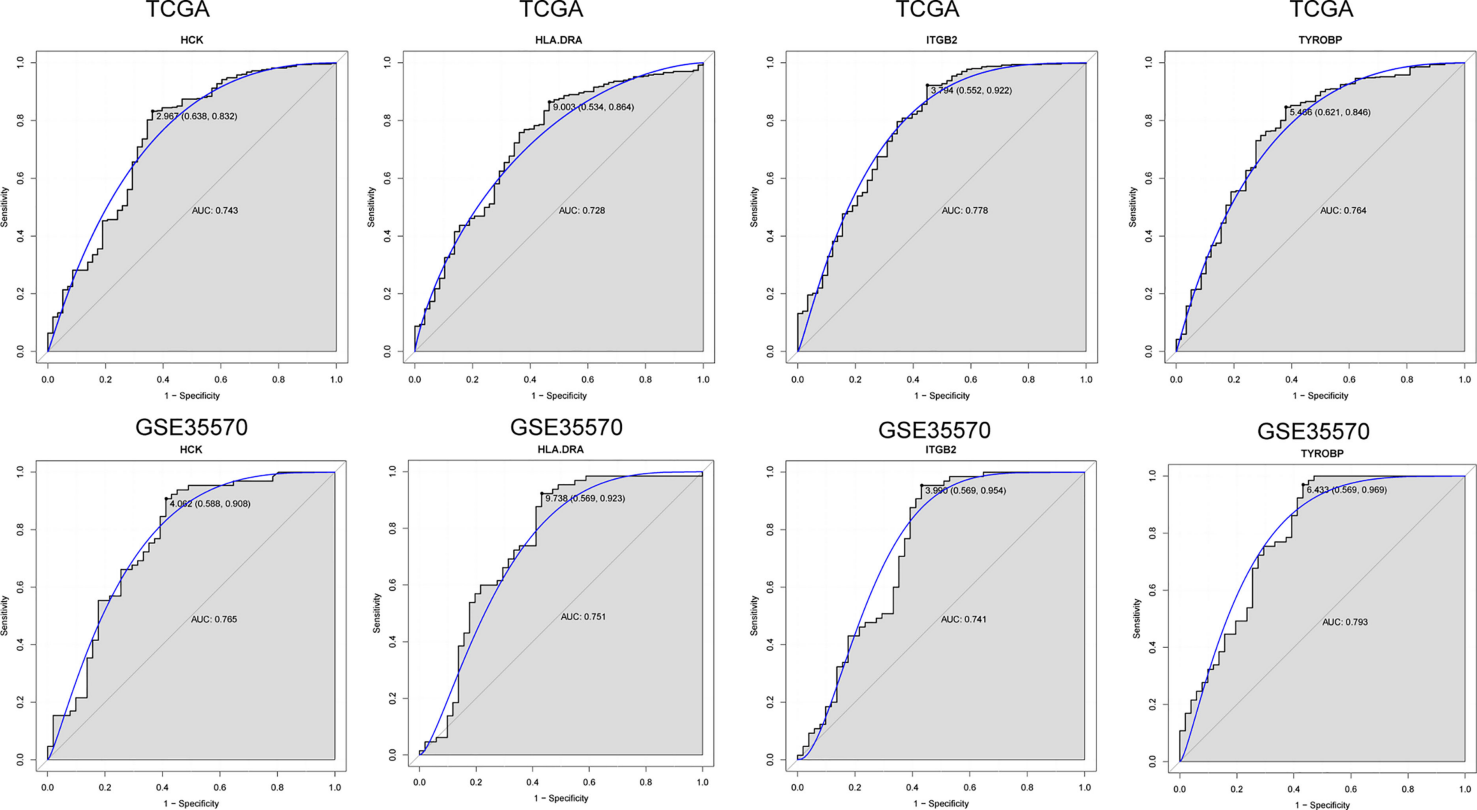
ROC analysis of 4 verified genes. The AUC was analyzed to evaluate the performance of each hub genes. The x-axis indicates 1-specificity and y-axis indicates sensitivity.

**Figure 9:**
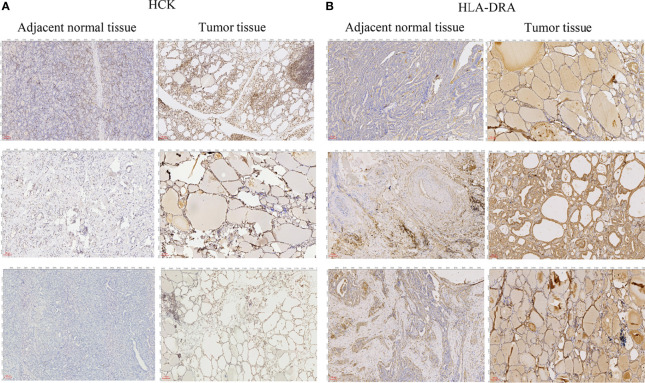
Immunohistochemical analysis of HCK **(A)** and HLA-DRA **(B)**. The length of scale bars in **(A, B)** is 100 μm.

To further verify the results of our bioinformatics analysis, the expression of 5 genes (C1QA, HCK, HLA-DRA, ITGB2 and TYROBP) were measured by quantitative real-time PCR in the tissue sample of patients with PTC and adjacent normal controls. Relative gene expression was analyzed using the 2^−ΔΔCt^ method. All primers used are shown in **Table 1**. As showed in **Figure 7D**, except for C1QA, the expression of HCK, HLA-DRA, ITGB2 and TYROBP was significantly up-regulated and was consistent with those of our bioinformatics analysis. Then, the diagnostic value of HCK, HLA-DRA, ITGB2 and TYROBP was assessed in TCGA and GSE35570 datasets. These results revealed that HCK, HLA-DRA, ITGB2 and TYROBP could distinguish PTC from adjacent normal controls (**Figure 8**). In the immunohistochemistry, the expression of HCK and HLA-DRA were positive correlated with the occurrence and development of PTC. The number and degree of cells staining positive for HCK and HLA-DRA genes were significantly increased in PTC tissues compared with adjacent normal tissues (**Figure 9**).

**Table 1:** Primer sequences used for quantitative real-time PCR.

Primer name	Primer sequence (5’ to 3’)
GAPDH-F (internal reference)	5-CTGGGCTACACTGAGCACC-3
GAPDH-R (internal reference)	5-AAGTGGTCGTTGAGGGCAATG-3
ACTB-F (internal reference)	5-GATCAAGATCATTGCTCCTCCT-3
ACTB-R (internal reference)	5-TACTCCTGCTTGCTGATCCA-3
TYROBP-F	5-ACTGAGACCGAGTCGCCTTAT-3
TYROBP-R	5-ATACGGCCTCTGTGTGTTGAG-3
C1QA-F	5-GAAATCTGCCTGTCCATCGT-3
C1QA-R	5-CCTCAGAGCCCTGGTAAATG-3
HLA-DRA-F	5-ATACTCCGATCACCAATGTACCT-3
HLA-DRA-R	5-GACTGTCTCTGACACTCCTGT-3
HCK-F	5-TCCGGGATAGCGAGACCAC-3
HCK-R	5-CCCGTCGTTCCCCTTCTTG-3
ITGB2-F	5-TGCGTCCTCTCTCAGGAGTG-3
ITGB2-R	5-GGTCCATGATGTCGTCAGCC-3

## Discussion

During past years, the incidence of PTC is on the rise worldwide. It is worth noting that PTC is often accompanied by hashimoto’s thyroiditis and the most familiar autoimmune thyroid disease (30). A large number of studies have shown that PTC with hashimoto’s thyroiditis is usually less invasive, which may be related to the infiltration of immune cells in tumor tissue (31). A number of immune-related parameters have been used to predict the occurrence and development of PTC patients, suggesting that different immune states have an important impact on the occurrence and development of PTC (32, 33). Previous studies have found that immune microenvironment may be related to differentiation of thyroid cancer (34). Signal transducer and activator of transcription 6 (STAT6) is significantly positively associated with immune infiltration of B cells, CD4 T cells, neutrophils, macrophages and dendritic cells of thyroid cancer (35). The abnormalities of CD8 cells and CD163 cells in the tumor microenvironment are related to the progression of PTC (36). The frequency of thyroperoxidase (TPO)- and thyroglobulin (Tg)-specific CD8 T cells in PTC patients is largely increased compared with healthy controls (37, 38). The reduced or absent of MHC class I and β2-microglobulin expression in PTC is associated with the reduced of tumor-infiltrating immune cells, which is an important immune escape mechanism (39). Protective immune cells such as resting mast cells, resting natural killer cells, plasma cells, and regulatory T cells are reduced in the children and young adults-PTC (40). These studies indicated that immune cell infiltration plays an important regulatory role in the pathogenesis and development of PTC. Therefore, systematically comprehending the infiltration level of immune cells in PTC microenvironment is of great value for developing new therapeutic strategies and targets for PTC.

Herein, we used TCGA datasets to display the level of immune cell infiltration and performed the weighted gene network construction. In addition, we applied GSE35570 dataset to analyze the level of Activated dendritic cells infiltration and verify the correlation between hub genes expression and the infiltration levels of Activated dendritic cells. Finally, the immunological and clinical characteristics of these candidate genes were verified using database and quantitative real-time PCR. We identified 14 hub genes whose expression correlated to the infiltration level of Activated dendritic cells, suggesting that these genes may be involved in the underlying mechanism of PTC progression. The positive correlation of the expression values of the 14 genes with the Activated dendritic cells infiltration level was validated in GSE35570 and GSE33630 datasets. Expression validation of 14 genes in TCGA dataset was performed, and results suggested that only 5 genes (C1QA, HCK, HLA-DRA, ITGB2 and TYROBP) were differentially expressed between PTC and adjacent normal groups. Similarly, expression level of these 5 genes was validated in GSE35570 dataset. Finally, quantitative real-time PCR results showed the expression trends of 5 genes were consistent with the results of TCGA and GEO. Among which, the differential expression of HCK, HLA-DRA, ITGB2 and TYROBP were statistically significant.

Hematopoietic cell kinase (HCK), a non-receptor tyrosine kinase, is predominantly expressed in myeloid cells, which involved in various cellular processes, including proliferation, apoptosis, differentiation migration, adhesion and immune cell activation (41, 42). HCK has been reported to be highly expressed in breast cancer and its expression level is associated with the prognosis of breast cancer patients, which is considered to be a new biomarker and therapeutic target for breast cancer (42). HCK participates in the progress of glioblastoma through TGFβ signaling-mediated epithelial mesenchymal transition (EMT), and may become a promising target for the treatment of glioblastoma (43). Abnormal expression of HCK plays a key role in the proliferation and survival of mantle cell lymphoma and the retention of malignant cells in the lymphatic microenvironment supporting growth and survival, thus promoting the occurrence of lymphoma (41). To date, to our knowledge, the study of HCK in PTC is still blank. Based on bioinformatics, our study found for the first time that HCK was highly expressed in PTC and related to infiltration level of activated dendritic cells. In addition, quantitative real-time PCR results also proved that HCK was highly expressed in PTC. Moreover, it had good diagnostic value for PTC. Therefore, we speculated that the involvement of HCK in the progression of PTC could be related to the infiltration level of Activated dendritic cells.

Human leukocyte antigen DR alpha chain (HLA-DRA) has been reported to be involved in the progression of a variety of cancers. For instance, the expression level of HLA-DRA gene is associated with poor prognosis of adrenal cortical tumors in children (44). HLA-DRA is reported to be a reliable biomarker for KIRC, which may play an important role in immunotherapy and improve prognosis (45). High expression of HLA-DRA is considered to be an immune factor that predicts prognosis and plays an immunomodulatory role in non-muscular and muscular-invasive bladder cancer (46). This is the first time we have provided evidence that HLA-DRA is highly expressed in PTC and related to infiltration level of activated dendritic cells. Furthermore, quantitative real-time PCR results also proved that HLA-DRA was highly expressed in PTC. Moreover, it had good diagnostic value for PTC. Thence, we believed that HLA-DRA was also an important immune factor in PTC. Further study of its mechanism may provide a new target for PTC immunotherapy.

Integrin beta2 (ITGB2), a subunit of integrin, accelerates leukocyte adhesion to endothelial cells, resulting in extravasation (46). ITGB2 regulates metabolic switch in cancer-associated fibroblasts through mitochondrial oxidative phosphorylation of NADH to promote oral squamous cell carcinoma proliferation (47). LncRNA ITGB2-AS1 facilitates the breast cancer cells migration and invasion *via* elevating ITGB2 (48). Tyrosine kinase binding protein (TYROBP), also known as DAP12, is highly expressed in various cancers and is related to tumor progression. For example, Wu et al. have found that TYROBP is a potential prognostic biomarker for clear cell renal cell carcinoma (49). Jiang et al. have reported that TYROBP is negatively correlated with the prognosis of gastric cancer and positively associated with the predictive biomarkers of gastric cancer immunotherapy (50). In our study, ITGB2 and TYROBP were significantly up-regulated in PTC and associated with infiltration level of activated dendritic cells, which were consistent with those of our bioinformatics analysis. Moreover, ITGB2 and TYROBP had good diagnostic value for PTC. However, the role of ITGB2 and TYROBP in PTC has not been revealed. Therefore, the study on the mechanism of ITGB2 and TYROBP in PTC may provide a theoretical basis for exploring the target of immunotherapy for PTC.

Previous studies have found that complement C1qA chain (C1QA) is associated with distant metastasis of breast cancer (51). The abnormal methylation of C1QA is also associated with the progression of thyroid cancer and is a potential biomarker (52). In osteosarcoma, the expression level of C1QA is positively correlated with patient survival time and levels of macrophage and CD8 cell in the tumor immune microenvironment (53). It has been reported that 3 human C1Q genes are closely bundled on chromosome 1 (C1QA-C1QC-C1QB) and their basal and IFNγ-stimulated expression, largely restricted to macrophages and dendritic cells (54). In this study, C1QA was abnormally expressed in PTC and correlated with Activated dendritic cells. Therefore, we speculate that C1QA may play an important regulatory role in the progression of PTC. In addition, the identification of C1QA also provides a potential target for PTC immunotherapy and a potential research direction for later research.

The KEGG pathway enrichment analysis revealed that most genes were mainly enriched in pathways including cytokine-cytokine receptor interaction, cell adhesion molecules (CAMs), phagosome and chemokine signaling pathway, which are typical immune-related signaling pathways. Some researchers have found that the cytokine-cytokine receptor interaction and cell adhesion molecules (CAMs) are related to the pathogenesis of PTC based on an immunogenomic landscape analysis (55, 56). The critical role of phagosome in microbial elimination and antigen presentation makes them essential for innate and adaptive immunity (57, 58). Chemokines induce normal physiological and immune responses by recruiting specific cell populations to the site of infection or malignancy (59). These studies further imply that immune mediation plays an important role in PTC. In this study, KEGG enrichment results may provide potential directions for further research on the molecular mechanism of PTC.

In conclusion, the main purpose of our study was to discover Activated dendritic cells related biomarkers of PTC based on WGCNA and SSGSEA analysis. A total of 14 hub genes were found to be associated with the infiltration level of Activated dendritic cells. Through verification in TCGA and GSE35570 datasets, C1QA, HCK, HLA-DRA, ITGB2 and TYROBP were identified as potential biomarkers for PTC immunotherapy. Quantitative real-time PCR showed that the expression of HCK, HLA-DRA, ITGB2 and TYROBP was all significantly up-regulated, which was consistent with our bioinformatics analysis. Finally, these 4 hub genes had diagnostic value to distinguish PTC and adjacent normal controls. Nevertheless, our study has certain limitations. Large sample sizes are needed to validate the current findings, and the role of these genes in PTC needs to be further studied. In addition, the validation set (GSE35570 dataset) includes post-Chernobyl PTC population. Therefore, the validation results may not be generalizable to the larger PTC population.

## Data Availability Statement

Publicly available datasets were analyzed in this study. This data can be found here: https://xena.ucsc.edu/ and GSE35570.

## Ethics Statement

The studies involving human participants were reviewed and approved by No. 960 Hospital of PLA Joint Logistics Support Force. The patients/participants provided their written informed consent to participate in this study.

## Author Contributions

BZ, ZJ and ML contributed to the conception of the study. JZ, and ZZ contributed the materials and performed the experiment. YQ and WQ performed the data analyses. BZ, ZJ and ML contributed significantly in writing the manuscript. All authors read and approved the final manuscript.

## Funding

This work was supported by the Shandong Province Medicine and Health Science and Technology Development Plan Project (Grants nos. 2018WS126), Taian Science and Technology Development Plan Project (Grant no. 2018NS0101) and Jinan Science and technology innovation project of “Research the Molecular Mechanism of lncRNA-miR-221-TFCP2L1 Mediated Invasion and Metastasis of Follicular Papillary Thyroid Carcinoma” (202019026).

## Conflict of Interest

The authors declare that the research was conducted in the absence of any commercial or financial relationships that could be construed as a potential conflict of interest.

## Publisher’s Note

All claims expressed in this article are solely those of the authors and do not necessarily represent those of their affiliated organizations, or those of the publisher, the editors and the reviewers. Any product that may be evaluated in this article, or claim that may be made by its manufacturer, is not guaranteed or endorsed by the publisher.

## Supplementary Material

Supplementary Figure 1 Gene co-expression networks in WGCNA. **(A)** The sample dendrogram and trait heatmap. **(B)** Analysis of the scale-free fit index and mean connectivity for various soft-thresholding powers (β).

Supplementary Figure 2 Identification of gene modules associated with the immune cell infiltration of PTC **(A)** A total of 10 genes modules were identified. **(B)** The dendrogram of all genes is clustered based on a dissimilarity measure. **(C)** The scatter plot shows the correlation between gene significance for PTC and module membership in black module. **(D)** Relationship between 14 hub genes expression and Activated dendritic cells infiltration level in GSE33630.

Supplementary Figure 3 The correlation between hub genes and Activated dendritic cells pre- and post-Chernobyl in the GSE35570 dataset **(A–E)** Correlation analysis of C1QA, HCK, HLA-DRA, ITGB2 and TYROBP expression and the infiltration level of activated dendritic cells post-Chernobyl in the GSE35570 dataset. **(F–J)** Correlation analysis of C1QA, HCK, HLA-DRA, ITGB2 and TYROBP expression and the infiltration level of activated dendritic cells pre-Chernobyl in the GSE35570 dataset.
