# Supplementary figures and images for: Identification of Transcriptional Pattern Related to Immune Cell Infiltration With Gene Co-Expression Network in Papillary Thyroid Cancer
Source: Front Endocrinol (Lausanne). 2022 Feb 4;13:721569. doi: 10.3389/fendo.2022.721569 (PMC8854657; doi:10.3389/fendo.2022.721569)

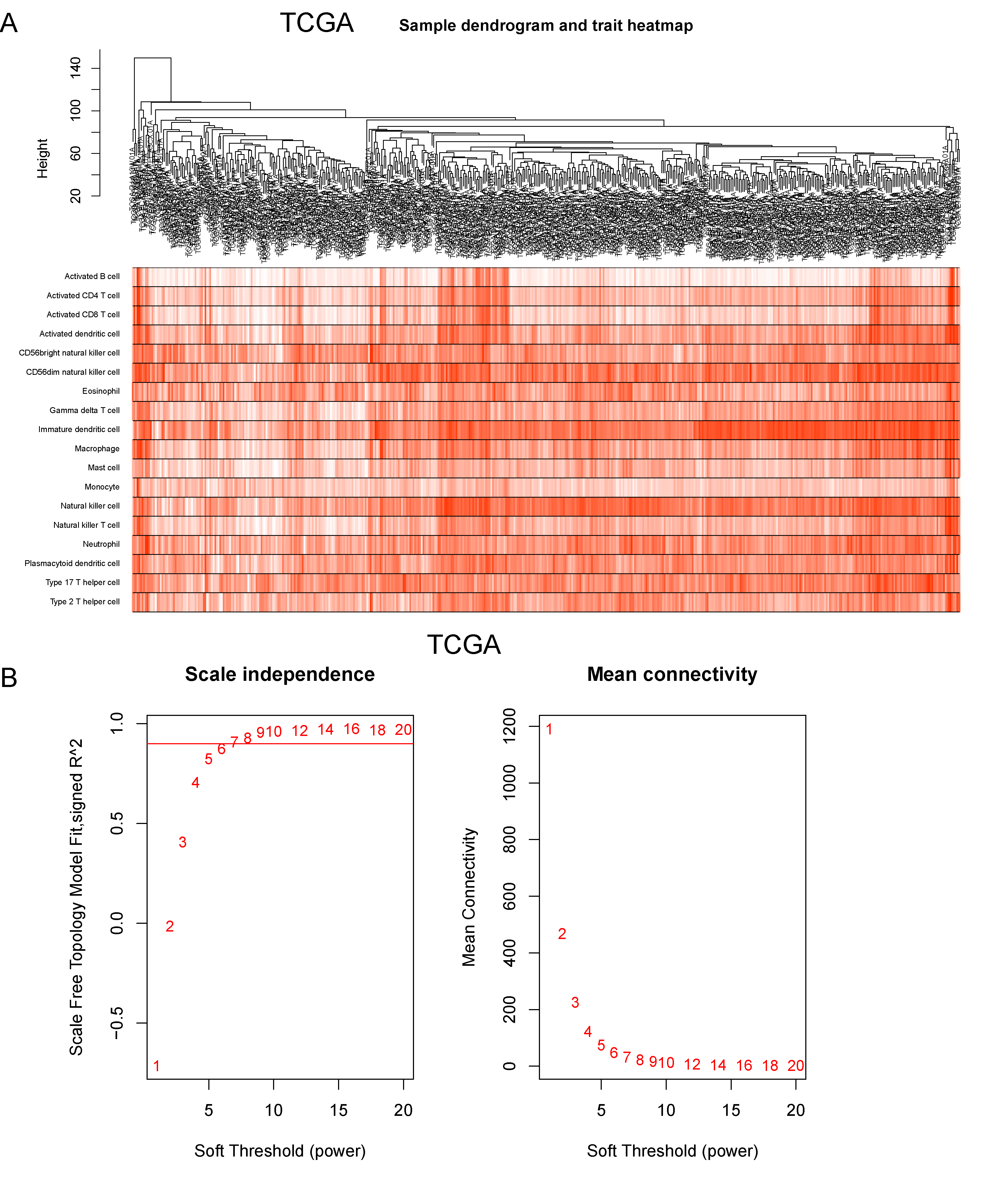

Supplement: Supplementary Figure 1 — Gene co-expression networks in WGCNA. (A) The sample dendrogram and trait heatmap. (B) Analysis of the scale-free fit index and mean connectivity for various soft-thresholding powers (β). [file Image_1.tif]

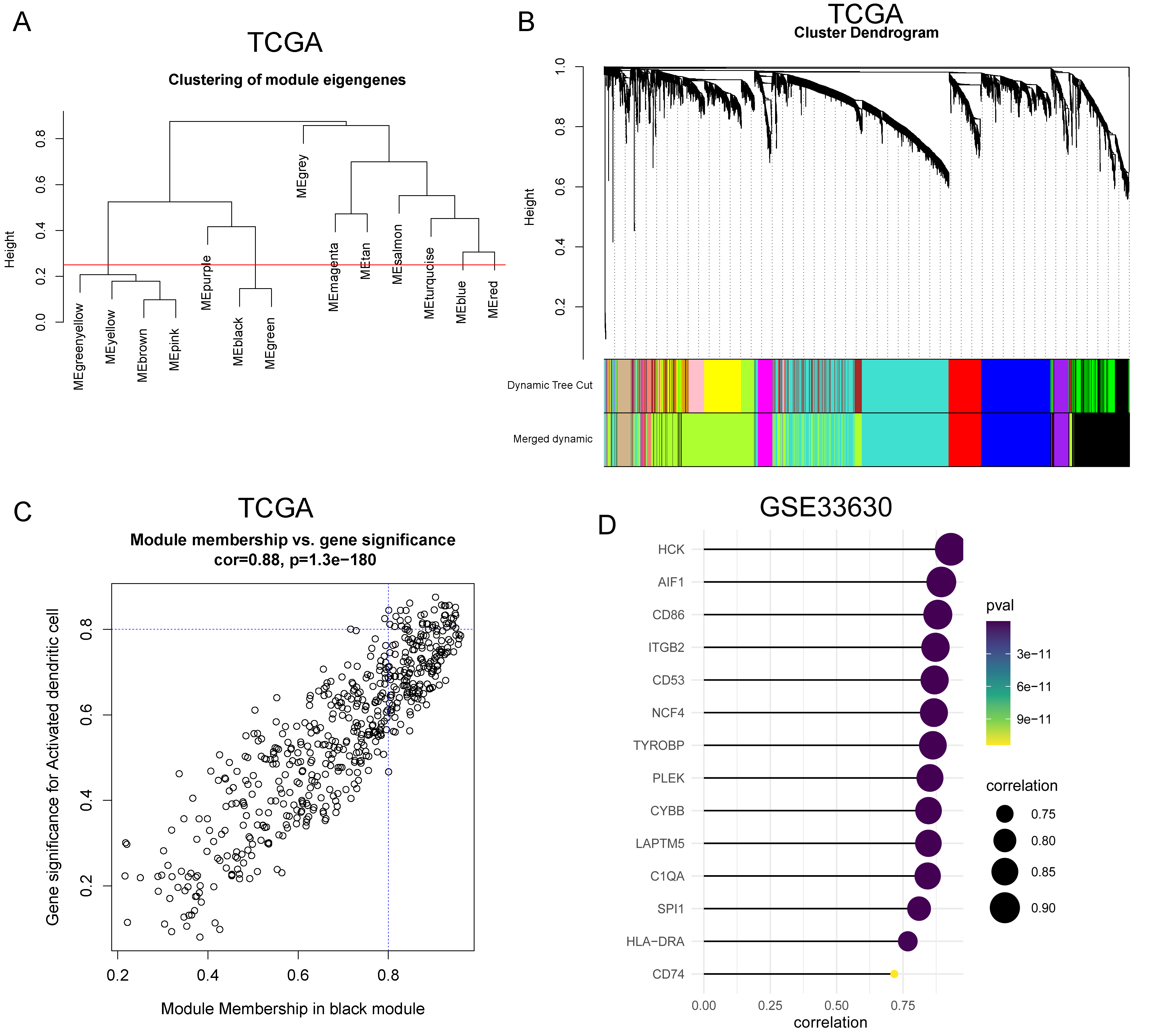

Supplement: Supplementary Figure 2 — Identification of gene modules associated with the immune cell infiltration of PTC (A) A total of 10 genes modules were identified. (B) The dendrogram of all genes is clustered based on a dissimilarity measure. (C) The scatter plot shows the correlation between gene significance for PTC and module membership in black module. (D) Relationship between 14 hub genes expression and Activated dendritic cells infiltration level in GSE33630. [file Image_2.tif]

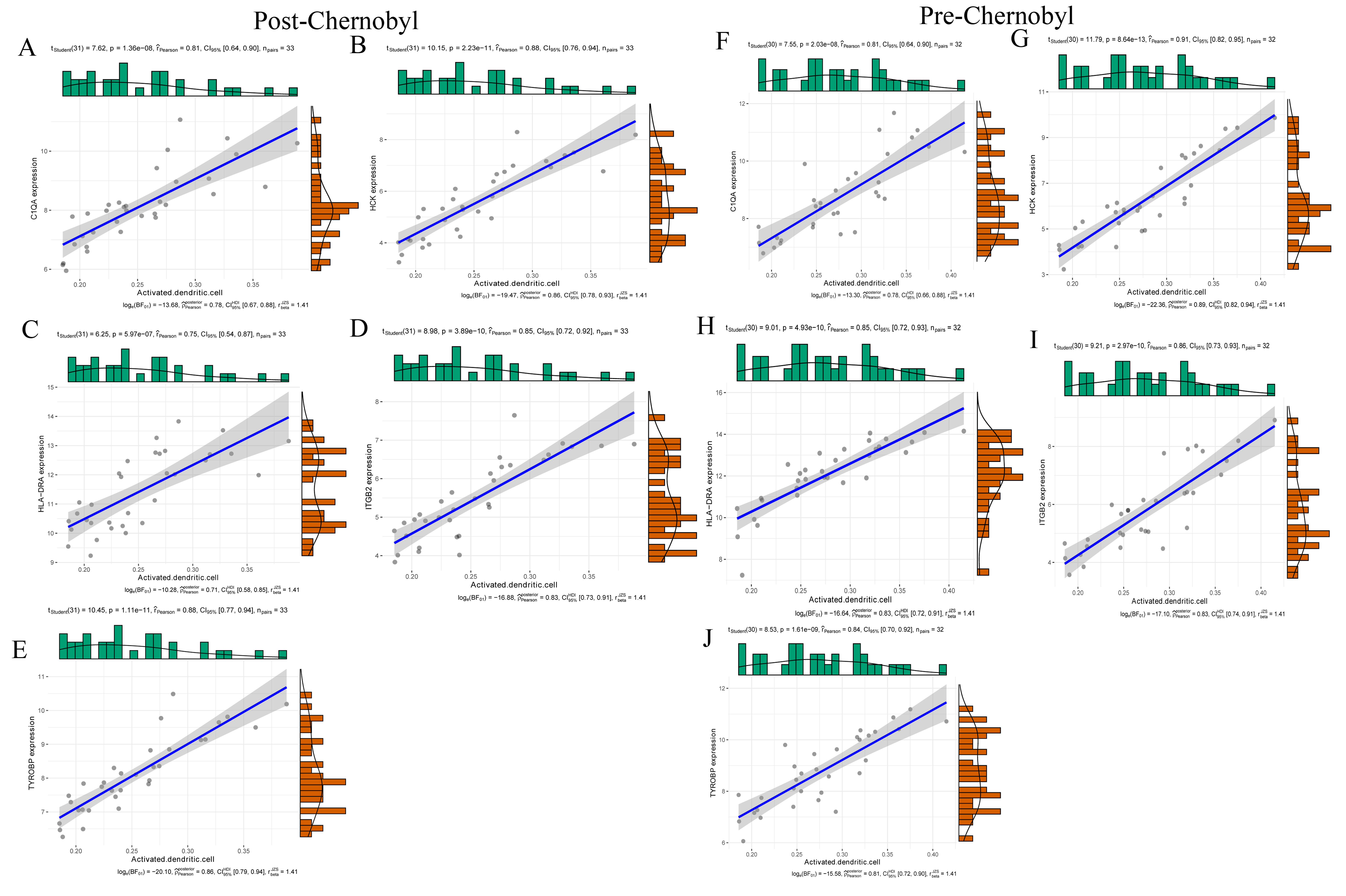

Supplement: Supplementary Figure 3 — The correlation between hub genes and Activated dendritic cells pre- and post-Chernobyl in the GSE35570 dataset (A–E) Correlation analysis of C1QA, HCK, HLA-DRA, ITGB2 and TYROBP expression and the infiltration level of activated dendritic cells post-Chernobyl in the GSE35570 dataset. (F–J) Correlation analysis of C1QA, HCK, HLA-DRA, ITGB2 and TYROBP expression and the infiltration level of activated dendritic cells pre-Chernobyl in the GSE35570 dataset. [file Image_3.tif]
